# High-Efficiency Multilevel Volume Diffraction Gratings inside Silicon

**DOI:** 10.1021/acsmaterialsau.3c00052

**Published:** 2023-10-13

**Authors:** Mehmet Bütün, Sueda Saylan, Rana Asgari Sabet, Onur Tokel

**Affiliations:** † Department of Physics, Bilkent University, Ankara 06800, Turkey; ‡ UNAM−National Nanotechnology Research Center, Bilkent University, Ankara 06800, Turkey

**Keywords:** 3D laser lithography, Talbot effect, in-chip, multilevel, diffraction gratings, microfabrication

## Abstract

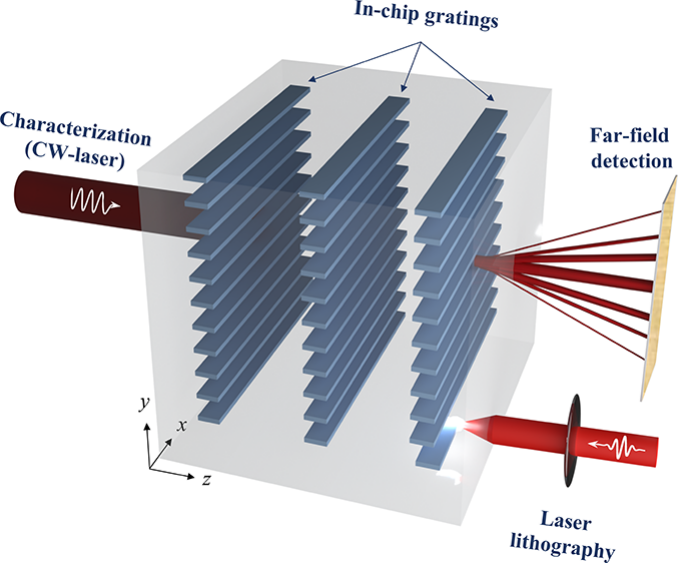

Silicon (Si)-based integrated photonics is considered to play a pivotal role in multiple emerging technologies, including telecommunications, quantum computing, and lab-chip systems. Diverse functionalities are either implemented on the wafer surface (“on-chip”) or recently within the wafer (“in-chip”) using laser lithography. However, the emerging depth degree of freedom has been exploited only for single-level devices in Si. Thus, monolithic and multilevel discrete functionality is missing within the bulk. Here, we report the creation of multilevel, high-efficiency diffraction gratings in Si using three-dimensional (3D) nonlinear laser lithography. To boost device performance within a given volume, we introduce the concept of effective field enhancement at half the Talbot distance, which exploits self-imaging onto discrete levels over an optical lattice. The novel approach enables multilevel gratings in Si with a record efficiency of 53%, measured at 1550 nm. Furthermore, we predict a diffraction efficiency approaching 100%, simply by increasing the number of levels. Such volumetric Si-photonic devices represent a significant advance toward 3D-integrated monolithic photonic chips.

## 1 Introduction

Diffractive optical elements (DOEs) are indispensable for numerous devices, such as holographic elements or spatial light modulators, which are widely used for beam shaping, 3D imaging, 3D projection, phase microscopy, and optical tweezing applications.^1−6^ Fabricating DOEs away from the surface, deep inside the material, provides a new degree of freedom toward increasing device efficiencies and also introducing new functionalities.^7^ Such systems would be resilient to surface damage and, more importantly, enable truly-3D optical systems through controlled modulation of optical index in 3D. Another goal would be monolithic integration of surface devices with subsurface systems for multilevel functionality.^8^ One example would be an architecture where input or output of on-chip components such as metasurfaces is modulated with 3D diffractive optics. The proposed volumetric elements can potentially find use as grating couplers in Si photonics. Diverse applications include spectroscopy, chemical and biological sensing, and multilevel integrated optics. Arguably the strongest candidate for creating such future systems is Si due to its importance for microelectronics, integrated photonics, photovoltaics, metamaterials, and terahertz systems. Thus, there is significant interest to explore the increased design freedom associated with the bulk of Si toward advanced in-chip functionality.

In recent years, 3D laser lithography has enabled the fabrication of photonic components in Si^8−14^ and various other materials.^15−18^ Here, we focus on 3D nonlinear laser lithography exploiting pulsed infrared lasers to create microstructures inside Si, with an optical index contrast on the order of 1 × 10^–3^ to 5 × 10^–4^.^8,9,19^ The magnitude of the index modulation limits the diffraction efficiency (DE) for practical device thickness values. For instance, the power ratio between the zeroth and first diffraction orders of Raman-Nath gratings fabricated with period Λ = 50 μm has been limited to 5–10%, measured at λ = 1310 nm wavelength, depending on the laser polarization.^19^ This corresponds to a modest 9–17% combined first order diffraction efficiency, which is defined as the total power of the first order beams normalized to the total power of all orders.

A promising direction to improve DE is to exploit the Talbot effect during the diffraction processes within the wafer. An analogous strategy has already been successfully implemented for glasses. For instance, using relatively low-index-contrast modifications, it has been possible to achieve enhanced DE by fabricating laser-written layers positioned at consecutive Talbot planes.^20^ The concept exploits a specific level-to-level separation corresponding to the separation of self-imaging planes.^20^ The increased efficiency is based on repeated energy coupling into the first order layer after layer. We exploit this approach for creating high-efficiency multilevel diffraction gratings buried in Si, which are fabricated with a period close to the fabrication wavelength. We further report a novel method for improved DE, which is based on optical lattice engineering using the Talbot effect. This novel concept reduces the level-to-level separation to just half the Talbot length, decreasing the use of volume by half for a given DE, and thus boosts the DE for practical device thicknesses.

Using this method, we experimentally demonstrate record first-order DE of 53% with 5-level gratings, compared to the 5% DE of single-level gratings of the same length (65 μm) and duty cycle (50%), measured at the telecommunication wavelength of λ = 1550 nm. We theoretically show that using the same set of parameters with an extended geometry, 7-level gratings can achieve >95% efficiency. Notably, by reducing the grating period toward characterization wavelength, we achieve higher diffraction angles, enabling rapid angular separation, in comparison to single-level volume gratings.^19^ Considered together, fabricated multilevel gratings and the reduced-volume self-imaging concept offer high-efficiency volumetric spatial control, which is a significant step toward 3D-integrated multilevel Si-photonics systems.^8^

## 2 Methods and Materials

### 2.1 Theoretical Modeling

**Figure 1:**
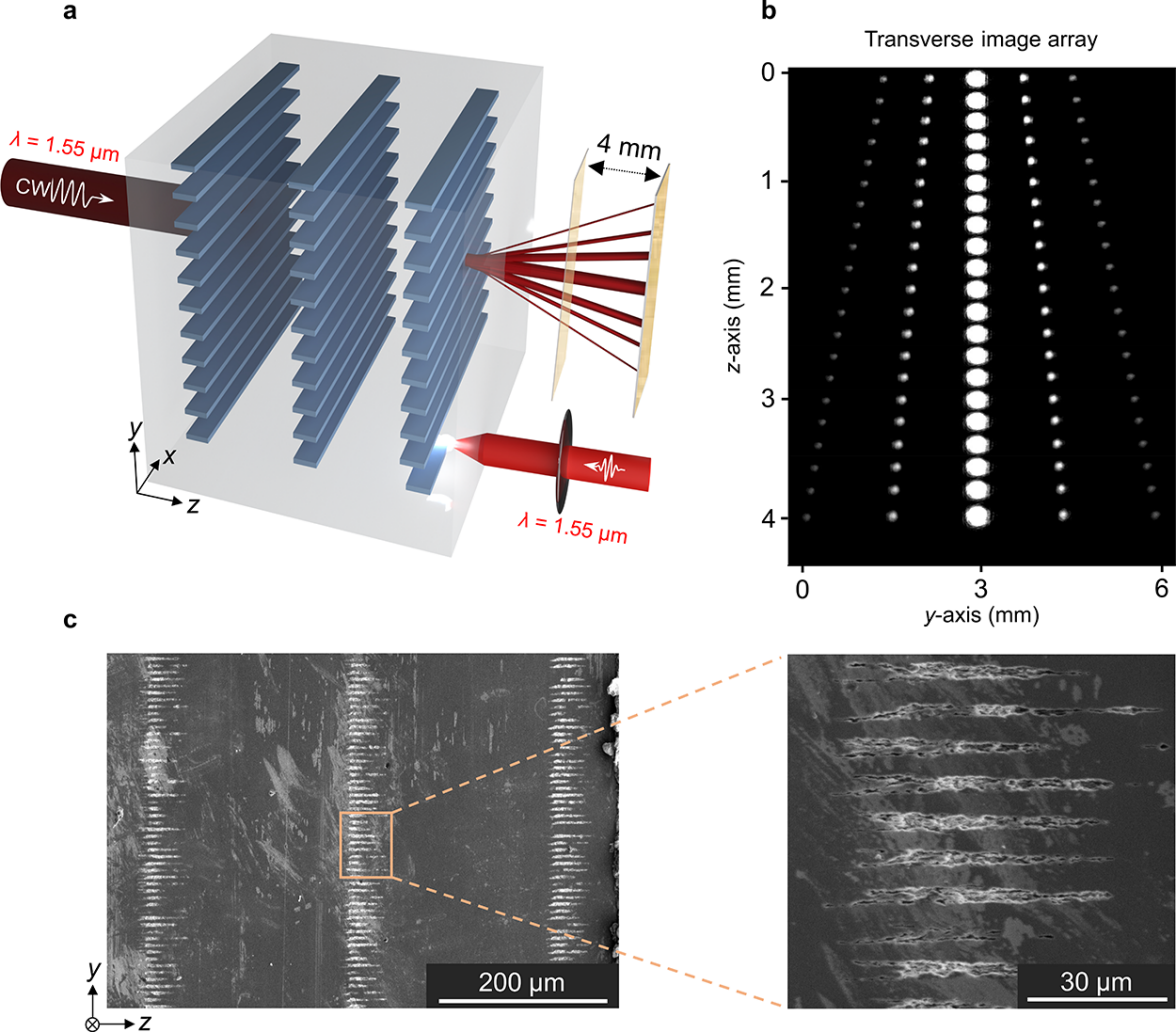
(a) Schematic representation of multilevel laser patterning inside Si and optical characterization of volume gratings. The laser beam on the right (λ = 1550 nm) is used for 3D patterning, while the beam on the left represents a continuous-wave laser (λ = 1550 nm) used for measuring the grating diffraction efficiency. (b) A representative array of 21 experimental images acquired on *x–y* planes shows the progress of diffraction orders along the *z*-axis. For ease of visualization, a laser wavelength of λ = 1310 nm is used for imaging. The data are recorded on 200 μm-separated planes over a distance of 4 mm. (c) The SEM image of the sample cross section confirms multilevel subsurface fabrication in Si. Inset: a close-up view from the middle level, revealed after brief chemical etching.

The near-field diffraction of single- and multilevel gratings (MLDGs) is simulated using finite-difference time-domain (FDTD) and finite element (FEM) methods using Ansys, Lumerical FDTD, and COMSOL solvers, respectively. The near-field intensity distributions obtained using 2D simulations are then projected to the far-field, which, in turn, are used for the diffraction efficiency calculations. Periodic boundary conditions are used for the *y* direction (Figure 1a), along which the optical index is modulated with an index contrast of |Δ*n*| = 2 × 10^–3^. This is the mean optical index modulation value estimated by evaluating the theoretical (obtained via FDTD) and experimental efficiency data from multiple single-level subsurface gratings. The refractive index of unmodified Si at λ = 1550 nm is taken as 3.48. In simulations, the laser-modified regions are approximated by rectangles on the *y–z* plane (Figure 1a). This geometry approximates the morphological data from laser-written areas analyzed by scanning electron microscopy (SEM). A normal-incidence plane wave is employed in simulations. The coordinate convention in Figure 1a is adopted throughout the paper.

### 2.2 Fabrication and Characterization

A home-built all-fiber master-oscillator power-amplifier (MOPA) system was used to fabricate the volumetric single- and multilevel diffraction gratings inside silicon (p-type, boron-doped, ⟨100⟩, 1–10 ohm-cm resistivity, 1–1.2 mm thick). The system operates at λ = 1550 nm and produces laser pulses in the range between 5 and 10 ns at 150 kHz repetition rate. A detailed description of the system can be found elsewhere.^9^ A three-axis high-resolution computer-controlled stage (Aerotech, ANT130Fi-XY, ANT95-L-Z) was used to scan the samples to create multilevel modifications. Individual grating levels are patterned with a single scan. A combination of a half-wave plate and a quarter-wave plate controls the laser polarization angle as well as the laser power on the sample. The gratings were patterned using a Gaussian-type laser beam with linear polarization, 1.5 μJ pulse energy, and 3 μm spot size inside the sample. The fabricated gratings have a projected surface area of 3 mm × 3 mm on the *x–y* plane.

An indium gallium arsenide (InGaAs) integrating sphere photodiode (Thorlabs, S146C) was used to measure the power of individual diffraction orders. As illustrated in Figure 1a, the gratings in Si were illuminated at normal incidence by a laser diode of wavelength λ = 1550 nm (Thorlabs, FPL1009S), focused to a spot size of ∼ 1 mm on the sample surface. The polarization of the beam was determined by a polarizing beam splitter (PBS) before the grating, while individual diffraction orders were selected by a pinhole positioned after the wafer. To acquire representative images of the diffraction orders, we illuminated the grating with a 1310 nm wavelength laser diode (Thorlabs, FPL1053S) and employed a CMOS camera (Thorlabs DCC1545M-USB 2.0) for imaging. The camera was mounted on a two-axis motorized translation stage, and diffraction was recorded at multiple planes separated by 200 μm from each other. The diffraction pattern of a multilevel, laterally aligned volumetric grating captured in this manner is given in Figure 1b.

SEM analysis was employed to identify various grating parameters such as periodicity, duty cycle, and length of grating levels (Figure 1c). Prior to the SEM analysis, the sample was diced and briefly etched (30 s) in an HF-based etchant (3 g of Cu(NO_3_)_2_·3H_2_O dissolved in a mixture of HF:HNO_3_:CH_3_COOH:H_2_O = 36:25:24:15 in volume) to reveal the cross section (Figure 1c). Statistical analysis was performed on over 60 measurements from the SEM analysis, showing a mean value of 35% for the duty cycle, along with the average period, line thickness, and level length values of 8 ± 0.25, 2.5 ± 0.8, and 65 ± 3.6 μm, respectively. These parameters were then used in the FDTD and FEM simulations.

## 3 Theoretical Formalism Based on the Talbot Self-Imaging Effect

A laser beam passing through a grating with normal incidence is separated into multiple diffracted beams. Immediately after the grating, a pattern forms due to interference in the outgoing field. It can be shown that the entire pattern is periodic along the direction of light propagation within the Talbot regime, with a period equal to the Talbot plane separation, *c*:^21^
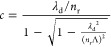
1

In this equation, λ_d_ is the free-space wavelength of the laser illuminating the grating, Λ is the grating period, and *n*_r_ is the background refractive index. An early experimental observation of this behavior was with sound waves, wherein two ultrasonic waves forming distinct grating levels were shown to establish periodicity in first-order diffraction, measured as a function of level-to-level separation.^22^ The insight afforded by the preceding concepts provides a basis for exploiting multiple levels positioned at precise separations toward enhanced optical device performance. However, such precise fabrication has been a long-standing challenge until laser lithography enabled the fabrication of 3D microstructures inside various materials. A notable example is multilevel gratings in fused silica, where each laser-written level is positioned at the Talbot planes.^20^ Such an architecture resulted in a significant diffraction efficiency enhancement.^20^

**Figure 2:**
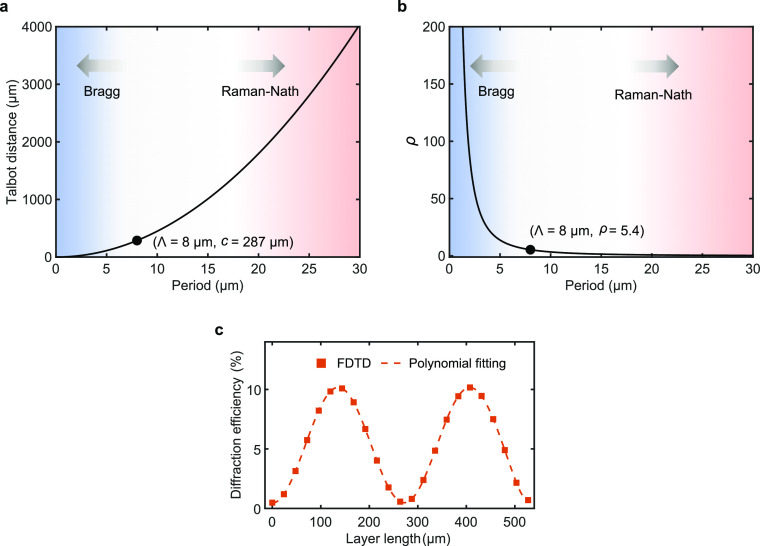
(a) Dependence of the Talbot distance on the grating period, Λ. Grating operating regimes are shown in the background with colors. (b) The parameter ρ is plotted against the grating period. Grating operates in the Raman-Nath regime for ρ ≤ 1, whereas large values of ρ (ρ ≫ 1) correspond to the Bragg regime. An intermediate regime between these two distinct cases is observed for 1 < ρ < 10. In experiments, ρ = 5.4 and *c* = 287 μm are chosen. (c) FDTD simulation shows the dependence of DE on the length of single-level grating with a period of 8 μm. The simulation assumes a normal incidence. A sinusoidal modulation of diffraction efficiency is observed, with a peak value of 10%. Duty cycle of 50% is assumed in panels (a)–(c).

We see from eq 1 that the separation between Talbot planes, which are also called self-imaging planes, strongly depends on the grating period, Λ. Decreasing the period decreases the Talbot distance, as given for Si in Figure 2a. This observation is instrumental in designing gratings with distinct levels centered at the Talbot self-imaging planes. Figure 2a also shows that the center-to-center distance between individual levels can be kept reasonably small. This will enable a commercially available 1 mm-thick Si wafer to accommodate high-efficiency gratings inside its bulk. For instance, a 4-level grating of Λ = 8 μm with a corresponding Talbot distance of 287 μm can be laser-patterned in Si to enhance the first-order DE by a factor of 4 to ∼40%. The corresponding value would be limited to ∼10% for a single-level grating of the same period, regardless of the grating layer length, *L* (Figure 2c). With these parameters and a large enough illumination beam (i.e., 1 mm diameter), the Talbot effect can be observed up to ∼1.8 cm. The FDTD computation in Figure 2c considers an index contrast of |Δ*n*| = 2 × 10^–3^ and 50% duty cycle (defined as the width of laser-modified lines divided by Λ).

Two important regimes are identified for the operation of gratings. Raman-Nath diffraction produces higher diffraction orders, while only one diffracted beam is produced in the Bragg regime, which occurs for near-Bragg incidence.^23^ In our approach, the lower limit for Λ is determined by the diffraction regime in which the device operates. Since the period in eq 1 applies to the near-field distribution resulting from the propagation of normal-incidence light,^24^ the period is chosen such that the grating does not operate in the Bragg regime. We have used the parameter ρ, defined as λ_d_^2^/Λ^2^*n*_r_Δ*n*, as a criterion for deciding which regime will apply.^25^ Values of ρ ≤ 1 correspond to the Raman-Nath regime, whereas large values of ρ (ρ ≫ 1) correspond to the Bragg regime operation. Further, the relative intensity coupled to higher order modes is on the order of 1/ρ^2^, so almost ideal Bragg behavior is obtained for ρ *>* 10. Thus, by variation of the grating period, a gradual transition between the Raman-Nath and Bragg regimes can be established. Based on the preceding considerations, a period of Λ = 8 μm is determined for our experiments to establish a balance between the requirements dictated by the diffraction regime and the requirement for a small distance between the levels of the grating (Figure 2a,b). The selected period corresponds to ρ = 5.4 such that the grating operates at the intermediate regime between the Bragg and Raman-Nath regimes. We also note that the method would not require any conceptual changes for adapting to different optical properties in the laser-written sections, such as modifications with a larger refractive index contrast.

## 4 Modeling and Experimental Results

**Figure 3:**
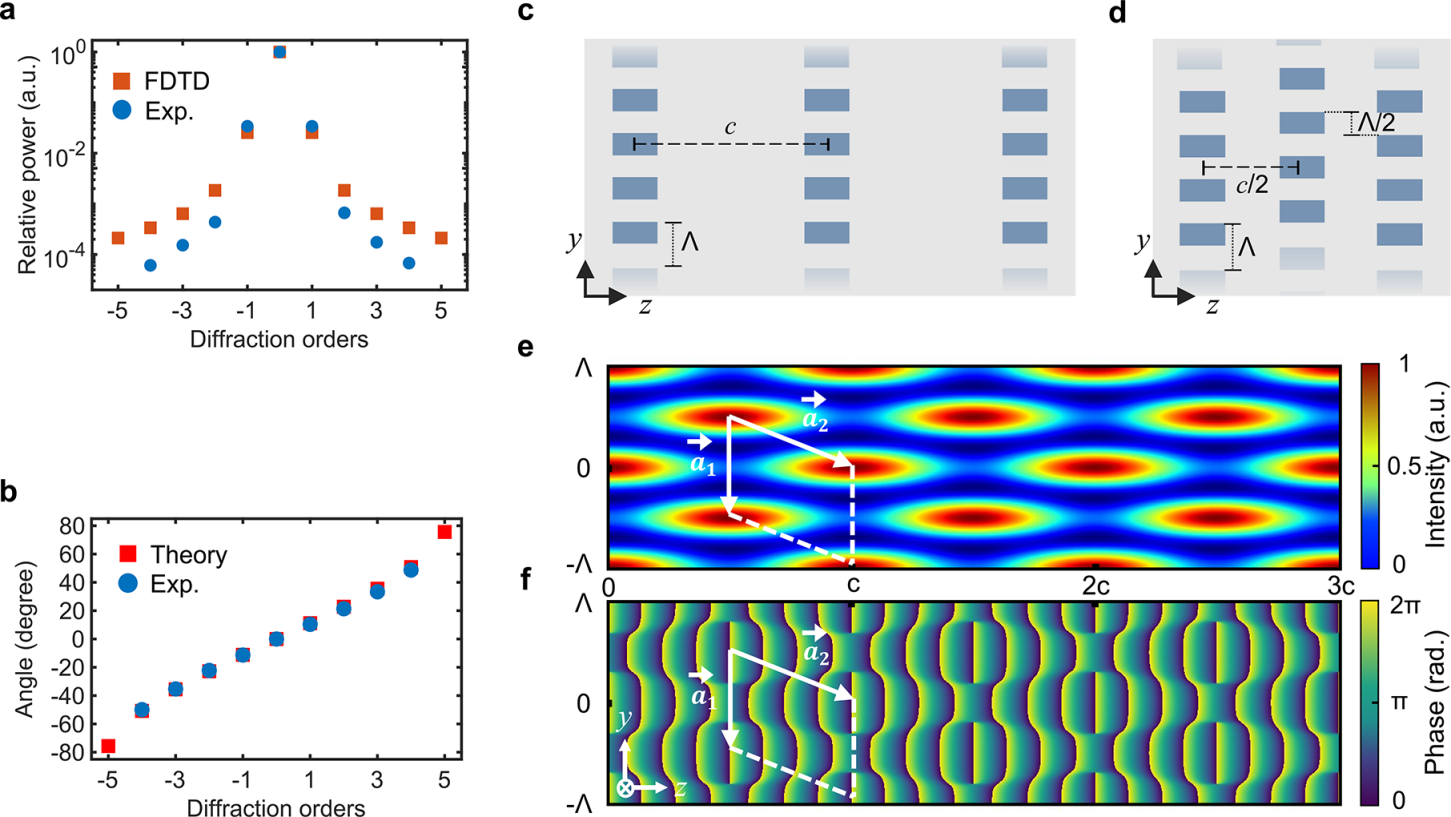
(a) Relative power, theoretical versus experimental, carried by the diffraction orders of a single-level grating created by using the parameters in Table 1. (b) Angular distribution of the diffraction orders from the same grating is determined by the grating equation and compared with measurements. (c, d) Schematic illustrations of the multilevel grating designs. The schematics show three levels along the *z* axis, but up to five levels have been fabricated in Si and reported in the paper. (c) Laterally aligned grating array design: levels are aligned with respect to each other and separated by the Talbot plane separation *c* in the *z* direction. (d) Proposed laterally shifted grating array design: levels are shifted by half period Λ/2 along the *y* axis, in relation to the levels before and after them and separated by *c*/2, half the Talbot plane separation, in the *z* direction. (e, f) Near-field intensity and phase distributions generated by interference of three beams that represent the 0 and ±1 orders of a 5-level grating with Λ = 3.2 μm. The relative power in the three orders is calculated by FDTD modeling. Higher diffraction orders can be ignored for the emergence of the Talbot self-imaging effect. The basis vectors of the optical lattice are also superposed over intensity and phase patterns (*c*: Talbot plane distance; Λ: grating period).

We start with experimental demonstration of single-level gratings and then move on to multilevel designs. Figure 3a,b shows theoretical and experimental values for the relative power and angular distributions of diffracted orders, evaluated for a single-level grating. The simulations employ the grating parameters given in Table 1, which are based on the SEM analysis of laser-written modifications. The theoretical diffraction efficiency at λ = 1550 nm is computed by projecting the near-field electric-field pattern obtained with the FDTD solver to the far-field. We observe very good agreement between the theory and experiment, indicating the accuracy of 3D laser lithography in fabricating in-chip diffraction gratings. These single-level gratings provide 5% combined first-order diffraction efficiency.

**Table 1:** Simulation Parameters

**property**	**symbol**	
characterization wavelength	λ	1.55 μm
background refractive index	*n*_r_	3.48
refractive index contrast	Δ*n*	2 × 10^–3^
width of modifications	*w*	2.8 μm
length of individual grating levels	*L*	70 μm
Talbot length	*c*	287 μm
grating period	Λ	8 μm
duty cycle	DC	35%

A significant increase in DE will be possible with two distinct multilevel grating designs. First, we theoretically and experimentally demonstrate that by a design involving an array of subsurface levels aligned with respect to each other and separated by the Talbot plane separation *c* in the direction of light propagation (Figure 3c), it is possible to create high-efficiency (39%) diffraction gratings in Si. Second, we propose an alternative design (Figure 3d) for achieving higher device performance with multilevel gratings for practical device thicknesses. We demonstrate that by shifting the levels by half the period (Λ/2) in relation to levels before and after them, while also reducing the interlevel distance to *c*/2 (Figure 3d), it is possible to achieve significantly improved diffraction efficiency in experiments (53%). Due to the reduced distance (*c*/2), these gratings reduce the volume requirement by half. The method also predicts significant DE enhancement by increasing the number of levels to seven (5% of single-level grating versus 85% of multilevel grating). As will be shown later, this advance is a direct consequence of enforcing the intensity and phase front distributions diffracted by the first level, through facilitating the patterns to be repeated and amplified at consecutive levels.

An important consideration for the preceding multilevel designs is the optical lattice pattern that emerges in the Talbot regime after the final level of a volume grating (Figure 3e,f). For the following analysis, a small grating period is selected (Λ = 3.2 μm) to keep the Talbot distance, *c,* small. The optical lattice is created with interference of the diffracted components carrying significant power (*m* = 0 and *m* = ±1); thus, higher diffraction orders may safely be ignored for a conceptual understanding. Figure 3e,f shows respectively the electric field intensity and phase distributions obtained with simultaneous free-space propagation of three such beams. The relative power carried by each order is assigned based on the relative power of 0 and ±1 orders of a 5-level grating evaluated with FDTD modeling. Thus, the optical lattices in Figure 3e,f capture the salient features of the near-field behavior following a high-efficiency multilevel grating. We note that similar lattice symmetry emerges for both multilevel designs, as well as for a single-level grating. We exploit this symmetry for the positioning of subsurface levels in Si. As seen from Figure 3e, one can travel between the maximum intensity points by translating with integer multiples of basis vectors  and , where  and . The self-consistent positioning of consecutive laser-written levels based on this translation symmetry preserves the optical lattice patterns and forms the basis of two designs (Figure 3c,d). We also note that at the maximum intensity transverse planes of Figure 3e, the phase front takes a square-like form, which will be relevant later (Figure 3f). Next, we consider the two multilevel designs (Figure 3c,d) in more detail, as well as the associated experimental results.

### 4.1 The Laterally Aligned Grating Array Design

**Figure 4:**
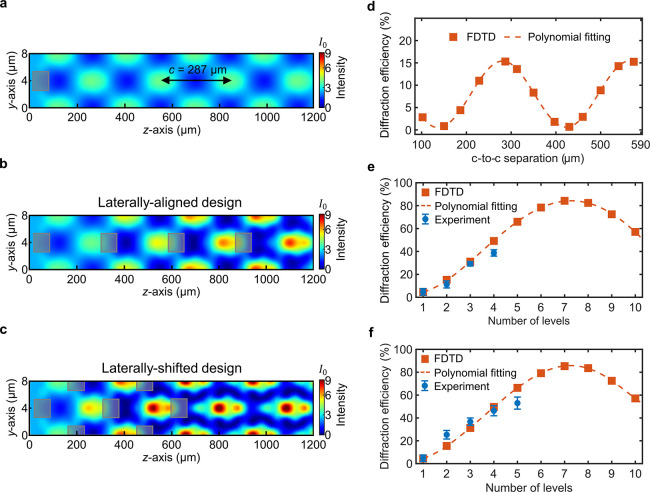
(a–c) Electric-field intensity distributions in the near-field for various single- and multilevel architectures. The outer black frames represent the 2D simulation area, while the inner rectangular blocks represent the laser-written regions, which form the distinct levels of the grating. The length of the simulation area along the *y* axis is equal to one period of the grating, which is repeated with the periodic boundary condition in simulations. The electric-field intensity distributions presented in panels (a)–(c) belong to single-level, laterally aligned 4-level, and laterally shifted 5-level gratings, respectively. (d) Combined first-order diffraction efficiency as a function of center-to-center (c-to-c) separation for a 2-level grating in Si. (e, f) Theoretical and experimental combined first-order efficiency values as a function of the number of distinct levels for the (e) laterally aligned and (f) laterally shifted grating designs in Si. All computations assume the grating parameters given in Table 1.

To illustrate the concept of this design, we start with single-level gratings in Si. Figure 4a shows the FDTD simulation of the electric-field intensity, where one already observes the self-imaging effect at consecutive high-intensity planes. These are separated by a Talbot distance *c* = 287 μm, which is equal to the analytical value calculated from eq 1.

We next investigated the effect of adding a second grating level, which is identical to the first level but translated along the *z* axis. The FDTD-based computation in Figure 4d shows the combined first-order DE as a function of the level-to-level separation for the 2-level grating. We observe that the optimal level-to-level separation is 287 μm. This value coincides precisely with the analytically and numerically computed Talbot distances *c* found for the preceding single-level grating. A further observation is that the theoretical diffraction efficiency drops to zero at the level-to-level separation values corresponding to *c*/2 and 3*c*/2, which is a manifestation of destructive interference of the diffracted beams.

Next, we show that the first-order efficiency can be significantly enhanced by an array of subsurface levels uniformly separated by the optimal distance found for the preceding 2-level grating, equal to the Talbot distance of *c* = 287 μm. This arrangement corresponds to the design in Figure 3c. Figure 4e demonstrates that, theoretically, around 50% combined first-order efficiency can be realized at an operating wavelength of 1550 nm by a 4-level grating, while the experimentally obtained efficiency (39 ± 2.8%) is close to the theory. The experimental efficiency is insensitive to polarization based on *s*- and *p*-polarized characterization experiments. Compared with the single-level efficiency of ∼5% (Figure 3a), the 4-level grating results in an approximately 8-fold enhancement. It is further observed from Figure 4e that the DE gradually increases as the number of levels increases, and DE reaches its peak value of 85% at seven levels.

The monotonic increase observed in the DE with the level count is reflected by the gradual increase in intensity after each consecutive Talbot plane level (Figure 4b) until the field eventually converges to the form given in Figure 3e,f. Further, the far-field projections from near-field planes at different locations inside the device indicate a uniform flow of power from the zeroth order to ±1 orders along the grating (Figure S1), concomitant with near-field intensity enhancements. This observation, combined with the near-field interference pattern of the zero and ±1 orders of a 5-level grating (Figure 3e), suggests that consecutive levels facilitate the propagation of ±1 orders while suppressing the zeroth order. It is worth noting that the theory predicts a sinusoidal dependence of DE on the number of levels,^20^ which indicates that DE will oscillate between a minimum and maximum as the number of levels varies. As shown in Figure 4e, the DE starts falling off at eight levels, following the sinusoidal trend. We further note that the coherence length of the laser is larger than the optical path length differences (OPDs) of the three main interfering beams for all levels, including the highest efficiency case of seven levels.

### 4.2 The Laterally Shifted Grating Array Design

In the previous section, we have shown an 8-fold enhancement in DE using a multilevel grating design, in which the levels are aligned in the *y* direction (Figure 3c). This section focuses on the performance of a new 3D architecture, where the individual grating levels are shifted laterally by half of the period Λ/2 in relation to the levels before and after them, which corresponds to the proposed design in Figure 3d. Here, we show that it is theoretically possible to realize gratings deep inside Si with 85% (35% duty cycle) and 95% (Figure S3, 50% duty cycle) first-order efficiencies and experimentally demonstrate a DE of 53 ± 5.3%, exploiting 3D nonlinear laser lithography in a Si wafer of only 1 mm in thickness (Figure 4f). The angular acceptance range of the gratings is found to be ±1° as defined by the full width at half-maximum of DE as a function of incidence angle obtained from FDTD modeling.

In comparison to the laterally aligned gratings, this novel design enables reducing the center-to-center distance between levels to half the Talbot distance and thus allows for a higher number of levels to be fabricated in the same volume. Although the approach pushes the DOE technology in a new direction, its theoretical background leverages the pioneering theoretical work,^24,26^ where it was shown that in the case of normal incidence, intensity distribution after a grating is repeated with a period of *c*/2 in the direction of light propagation, while it is displaced by Λ/2 in the direction of the grating period. That is, the patterns observed at the planes located within 0 ≤ *Z* ≤ 1/2 are visible again at the planes within 1/2 ≤ *Z* ≤ 1, but they are shifted by Λ/2 in relation to the grating.^24^ Here, *Z* is a unitless spatial parameter defined as *Z* = (*z – L*)/*c*, where *z* is the distance along the axis of propagation, *L* is the grating length, and *c* is the Talbot distance as defined in eq 1.

The highest experimental DE of 53% in Figure 4f was created with five levels. The experimental DE values (blue circles) in Figure 4f start to deviate at five levels from the values predicted by FDTD modeling (orange squares). We ascribe this to the sensitivity of the design to the lateral position of consecutive levels (Figure S2). Minor deviations from the ideal Λ/2 value that falls within the repeatability of the translational stage will cause a measurable decrease in efficiency. Since this is a cumulative effect, this design requires highly repeatable stages for increased efficiency devices. A less prominent reason for the deviation in later levels is assuming an ideal rectangular shape in the simulation, whereas the morphology observed in SEM analysis is not a perfect rectangle (Figure 1c). The thickness is also not perfectly uniform. Measurements indicate a line thickness of 2.5 ± 0.8 μm of the laser-modified regions after brief chemical etching (Figure 1c), whereas a constant value of 2.8 μm is assumed in the simulations, acquired from measurements of unetched gratings. The effects of a slightly varying line form is potentially cumulative and may cause the deviation observed in an increased number of levels. Fortunately, this may be addressed by emerging methods employing spatially shaped beams, improving the uniformity and resolution of fabrication in Si.^27−29^

## 5 Conclusions

Using three-dimensional nonlinear laser lithography, we proposed and implemented two distinct architectures for multilevel grating fabrication in Si. These exploit self-consistent field enhancement based on the Talbot effect. The first design uses laterally aligned subsurface levels and experimentally yields 39% DE with 4-level gratings. The second design, which is based on a laterally shifted level architecture, achieves the same performance but within a substantially smaller volume by reducing the distance between grating levels to just half the Talbot distance. Thus, the latter design fits more levels within the same wafer and achieves a record diffraction efficiency of 53% measured with 5-level gratings. Further, our simulations indicate that 95% efficiency is possible with 7-level gratings (50% duty cycle), which may be created given enough volume in Si or translational stages with high repeatability. The experimentally demonstrated diffractive optical elements correspond to creating functionality emerging from the interaction of distinct levels within Si and constitute a significant step toward multilevel, multifunctional integration inside Si.

## Supporting Information Available

Near-field and far-field intensity patterns as a function of number of levels in multilevel grating designs; sensitivity of diffraction efficiency to fabrication error in lateral shift; theoretical diffraction efficiency with 50% duty cycle (PDF)

## Abbreviations

c-to-c: center-to-center
DE: diffraction efficiency
