# Supplementary material for: High-Efficiency Multilevel Volume Diffraction Gratings inside Silicon
Source: ACS Mater Au. 2023 Oct 13;3(6):727–33. doi: 10.1021/acsmaterialsau.3c00052 (PMC10636781; doi:10.1021/acsmaterialsau.3c00052)
Supplement: Supplementary file 1 — mg3c00052_si_001.pdf [file mg3c00052_si_001.pdf]

Supporting Information

High-Efficiency Multi-Level Volume Diffraction Gratings Inside Silicon

Mehmet Bütün, Sueda Saylan, Rana Asgari Sabet, Onur Tokel*

Department of Physics, Bilkent University, 06800 Ankara, Turkey

UNAM - National Nanotechnology Research Center, Bilkent University, 06800 Ankara, Turkey

* Corresponding author, E-mail: otokel@bilkent.edu.tr

1. Near-field and far-field intensity patterns as a function of number of levels in multi-level grating designs

The near-field patterns (Figure S1, colormaps) for the laterally-aligned and laterally-shifted architectures are evaluated at the indicated positions and then projected to far-field in order to calculate the relative power carried the corresponding colormaps. As expected, the zeroth order carries all the power just before the grating at $z = 0$. As the position marker moves along the z direction, the number of subsurface levels increases and gradually a higher portion of the incident power is transferred from the zeroth order to the ±1 orders. The transfer of power to the ±1 orders is shown for the laterally-aligned 4-level system (Figure S1,a). Similarly, for the laterally-shifted design only ~33 % of the total power is carried by the zeroth order after five levels (Figure S1,b).

In parallel, the phase computed at transverse planes after each grating level is shown underneath the colormaps, which changes from being flat at $z = 0$ to being square-like at the output of 4- or 5-layer gratings. The square-like phase is the manifestation of two-beam interference [1]. As the light propagates through the multi-layer grating, the zeroth order is almost suppressed and ±1 orders gain power. As a result, the interference of only ±1 orders remains, which creates the expected square-shaped phase. This pattern converges to the ideal case approximated in Figure 3f, Manuscript.

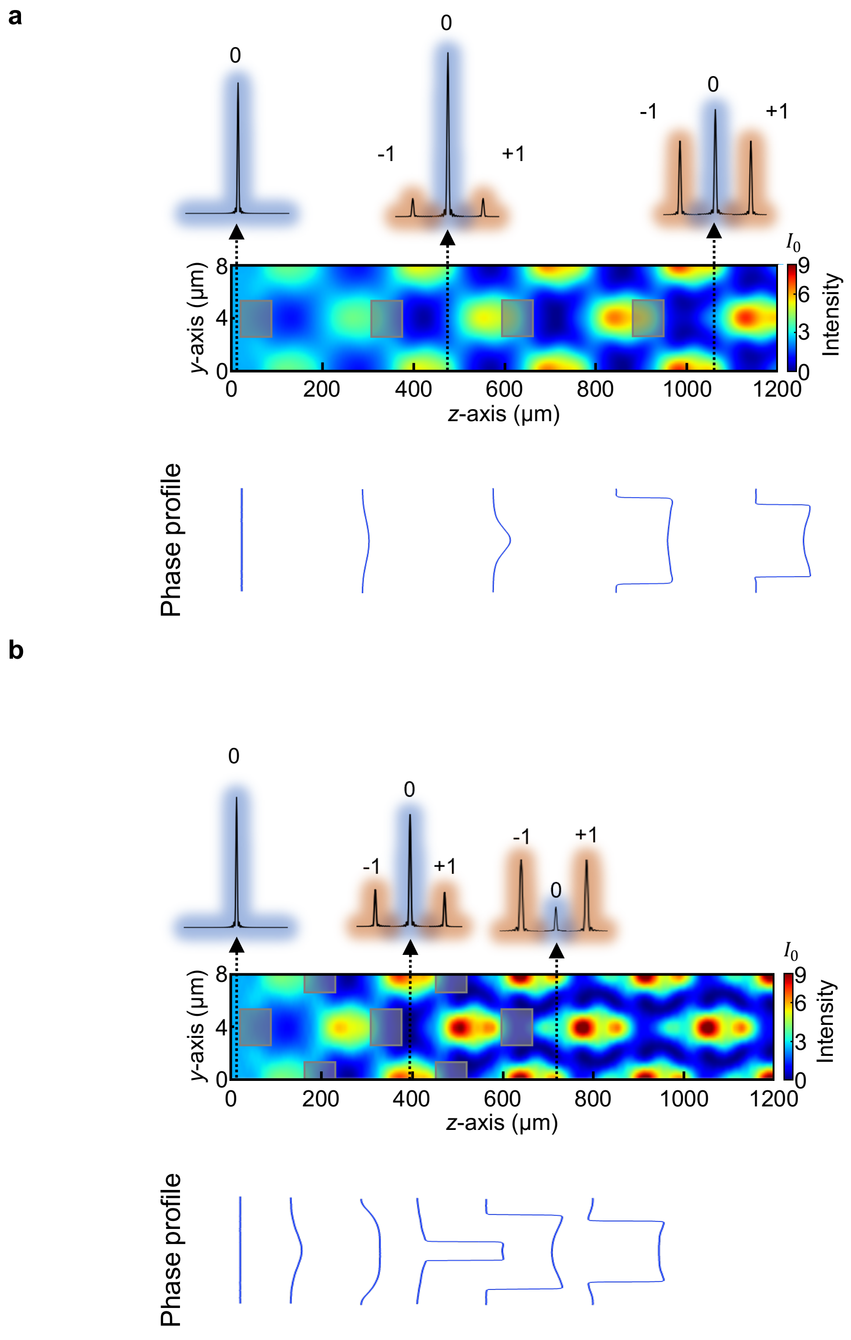

Figure S1. The electric-field intensity distributions in the near field for (a) 4-layer laterally-aligned grating, and (b) 5-layer laterally-shifted grating. The relative power in 0 and ±1 diffraction orders are shown above the colormaps at three different locations along the grating, which are proportional to the area under the curves. As the light propagates, the transfer of power from zeroth order to ±1 orders is notable level after level. Simultaneously, the phase evolves from a flat phase shown on the left to a square-like profile shown on the right, due to interference of remaining power in the ±1 orders.

2. Laterally-shifted design: Sensitivity of diffraction efficiency to fabrication error in lateral shift

Figure S2 shows the lateral-shift sensitivity of diffraction efficiency in a 2-layer laterally-shifted grating design. Any deviation from the ideal lateral shift of 4 μm for the given design is expected to reduce the efficiency.

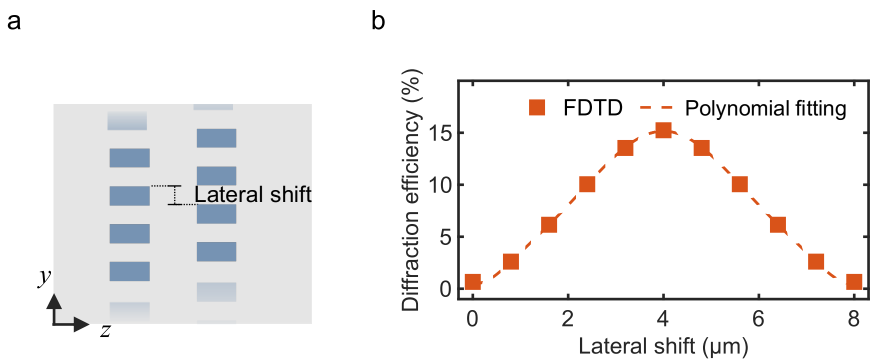

Figure S2. (a) Schematic of the laterally-shifted grating design in Si with a grating period of 8 μm and levels shifted with a center-to-center distance of 143.5 μm. (b) The lateral-shift dependence of diffraction efficiency in a 2-level laterally-shifted grating with 35% duty cycle.

3. Laterally-shifted design: Theoretical diffraction efficiency with 50% duty cycle

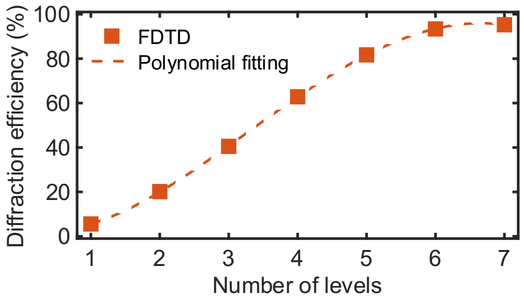

Figure S3. Theoretical combined first-order efficiency as a function of the number of levels for the laterally-shifted grating with 50% duty cycle.

References

[1] Kim, M.-S.; Scharf, T.; Menzel, C.; Rockstuhl, C.; Herzig, H. P. Talbot Images of Wavelength-Scale Amplitude Gratings. *Opt. Express* **2012**, *20* (5), 4903–4920. https://doi.org/10.1364/OE.20.004903.
